# A study on the characteristics and influencing factors of public sports service demand among the population of rural older adults in China

**DOI:** 10.3389/fpubh.2025.1613449

**Published:** 2025-07-08

**Authors:** Hai-pei Zhang, Cong-hui He, Wen-yun Lu

**Affiliations:** ^1^School of Economics and Management, Shanghai University of Sport, Shanghai, China; ^2^School of Physical Education, Shanghai University of Sport, Shanghai, China

**Keywords:** rural older adults, public sports service demand, influencing factors, satisfaction, practices in China

## Abstract

**Background:**

With the continuous deepening of China’s aging population, the high prevalence of chronic diseases, high disability rate and significant medical expenses among older adults have brought huge burdens to individuals, families and the entire social development. Compared with urban areas, the aging population in rural areas is severe and the population’s health status is worse. Therefore, how to scientifically address the issue of population aging in rural areas and minimize the negative effects of aging has become a focus of common concern in all sectors of society. As an important non-medical health intervention, exercise has been proven effective by scientific research and is also a common experience of developed countries in actively responding to the issue of population aging. However, the traditional one-way government supply model of rural public products has led to inefficient or ineffective supply of public sports services for a long time, making it difficult to meet the growing personalized needs of rural older adults. Therefore, a comprehensive understanding of the actual demands of rural older adults for public sports services, clarifying their demand types, structural characteristics and influencing factors, is the prerequisite for achieving an effective supply of public sports services for rural older adults and promoting their regular participation in sports.

**Methods:**

To comprehensively understand the demand situation for public sports services for rural older adults, a sociological survey was conducted on the demand characteristics, influencing factors and satisfaction of public sports services for rural older adults aged 50 and above living in townships, corresponding towns and villages in the eastern, central and western regions of China. The research methods included a literature review, questionnaire survey, interview survey and statistical analysis.

**Results:**

Rural older adults have the most urgent demand for establishing sports fitness organizations, improving sports exercise guidance services and strengthening the construction of elderly sports systems; the demand for public sports services among rural older adults is influenced by multiple factors such as demographic characteristics, health status, individual lifestyle and sports exercise cognition level; the overall utilization rate of public sports services for rural older adults is low, and their satisfaction is at a medium level.

**Conclusion:**

To meet the public sports service demands of rural older adults, in the future, it is still necessary to enhance the demand subject consciousness of rural older adults, clarify the subjective demand picture of rural older adults, and improve the quality of public sports services for rural older adults.

## 1 Introduction

Since the 21st century, the number of people aged 60 and over has been growing at an unprecedented rate globally, particularly in developing countries. The increase in population aging has far-reaching implications for countries around the world in many areas, including economic development, social structure, and public health. As one of the world’s most populous countries, China leads the world in the scale of its population of rural older adults. With the continuous deepening of aging, health issues among older adults have garnered increasing attention from society (1). Compared to urban areas, rural regions in China experience higher levels of aging, with poorer health conditions among older adults. Lack of health knowledge and fitness methods is widespread (2). From an international perspective, the fundamental approach to addressing these problems is to fully leverage the positive role of sports in the process of population aging (3), gain a broad understanding of the needs for public sports services among older adults (4), and design corresponding service products to enhance the supply level and quality of public sports services, laying the foundation for healthy aging in rural areas. Currently, research on the demand for public sports services is primarily focused on urban communities (5, 6), with few studies examining the current status and influencing factors of public sports service needs from the perspective of older adults. Therefore, our study aims to systematically analyze the characteristics and influencing factors of public sports service demand among rural older adults in China by absorbing and drawing on the latest international research findings in the fields of older adult sports and public services to provide policy recommendations for global older adult sports or public sports.

## 2 Research subjects and methods

### 2.1 Research subjects

According to the needs of the study, the demand for public sports services among rural older adults was taken as the object of the study. With regard to the definition of the age of older adults, the World Assembly on Aging, convened by the United Nations in 1982, stated that “persons aged 60 years and over are considered to be older adults.” Some foreign studies on geriatric sports define older adults as over 65 years old, some as over 60 years old, and some as over 50 years old. Since the average life expectancy of older adults in rural areas is 12 years shorter than in urban areas (7), the onset of chronic diseases among older adults in rural areas mostly occurs at the age of 50. Therefore, from the perspective of early intervention in physical activity behaviors of older adults, our study defined the age of rural older adults as 50 years and above. In summary, the respondents of our study were people aged 50 and above living in townships, corresponding towns, and villages.

### 2.2 Method

#### 2.2.1 Questionnaire

The research was conducted using a literature review, questionnaire survey, interview survey, and statistical analysis. Both stratified and graded random sampling and simple random sampling methods were employed for the sampling process. The questionnaire on the demand for public sports services among rural older adults was distributed to older adults residing in rural areas of Fuding City (Fujian Province in the eastern region), Shaoyang County (Hunan Province in the central region), and Guanghan City (Sichuan Province in the western region). Investigators conducted door-to-door surveys with the questionnaires and assisted respondents in completing the forms through interviews. A total of 1,800 questionnaires were distributed in our study. Moreover, 133 invalid questionnaires were excluded, leaving 1,667 valid questionnaires with an effective recovery rate of 92.6%. The sample shows that in terms of sex, there were 906 male participants (54.35%), slightly higher than the number of female participants. In terms of age, the sample was predominantly composed of young older adults (under 70 years old), accounting for 70.12% of the total sample. In terms of education, more than 70% of older adults in the sample had an education level of “primary school and below.” In terms of income, the largest group was those with “general” economic conditions, numbering 1,047 people (62.81%), while those with “more difficult” economic conditions were relatively few, and those belonging to “affluent and above” families were the smallest group. In terms of marital status, the proportion of older adults with spouses (74.09%) was much higher than that of older adults without spouses. In terms of regional structure, the sample distribution was relatively balanced, with 437 people (26.24%) from the eastern region, 507 people (30.44%) from the central region, and 723 people (43.32%) from the western region. Overall, the population structure of the sample is relatively consistent with the actual living conditions of rural older adults, and the sample size in the eastern, central, and western regions is appropriate and uniform, making it highly representative.

#### 2.2.2 Univariate analysis

According to the Shapiro–Wilk test, continuous variables with a normal distribution are expressed as mean ± standard deviation, while variables without a normal distribution are expressed as median (25th and 75th percentiles). The categorical variable is represented as the number of cases. This study uses multiple imputations to handle missing continuous data. Independent sample t-test is used for inter-group comparison of continuous variables, while the chi-square test is used for categorical variables.

#### 2.2.3 Binary logistic regression analysis

To further explore the key factors associated with the specific demand for public sports services among rural older adults, this study employed binary logistic regression. The basic principle of this method assumes that the dependent variable Y follows a binomial distribution, taking values of 0 or 1, where *π* (Y = 1) represents the probability of the event occurring (Y = 1). For a set of independent variables denoted as x₁, x₂, …, x_n_, the logistic regression model is expressed as follows:


$$\pi =P(Y=1/X_{1}=x_{1},\ldots X_{n}=x_{n})=\frac{e^{\beta_{0}+\beta_{1}x_{1}+\beta_{2}x_{2}+\ldots \beta_{n}x_{n}}}{1+e^{\beta_{0}+\beta_{1}x_{1}+\beta_{2}x_{2}+\ldots \beta_{n}x_{n}}}$$

In a general regression model with a single independent variable, the relationship between the independent and dependent variables is linear. However, in binary logistic regression, the relationship between the independent variable and the probability *π* (Y = 1) follows an S-shaped (sigmoid) curve.

In this type of model, the maximum likelihood estimation (MLE) method is used to calculate the regression coefficients. The model output includes odds ratios (OR), which reflect the strength and direction of association between the binary outcome and each independent variable. Substituting the odds ratio into the regression model, the left-hand side of the equation is the logit transformation of the probability *π* (Y = 1), rather than the binary outcome Y itself:


$$\begin{array}{l} \log\;\text{it}[\pi (Y=1)]=\ln[\frac{\pi (Y=1)}{1-\pi (Y=1)}]=\ln(\hat{o})\\ =\beta_{0}+\beta_{1}x_{1}+\beta_{2}x_{2}+\ldots \beta_{n}x_{n} \end{array}$$

Each coefficient βⱼ represents the average change in the log odds of the outcome associated with a one-unit increase in independent variable Xⱼ, holding all other variables constant. In practical applications, the interpretation of the odds ratio is of primary interest. The odds ratio for a given independent variable Xⱼ can be used to evaluate the magnitude of its association with the dependent variable.


$$O\hat{R}=\exp(\beta_{j})$$

$$\text{OR}=\hat{o}=\exp(\beta_{0}+\beta_{1}x_{1}+\beta_{2}x_{2}+\cdots +\beta_{n}x_{n})$$

Moreover, to control the risk of Type I errors resulting from multiple significance tests, we adjusted the original *p*-values using the Bonferroni correction method. All statistical analyses were performed using SPSS 27.0.

## 3 Concept definition

### 3.1 Public sports services for rural older adults

The definition of “public sports services for rural older adults” serves as the logical starting point for our study, grounded in the broader notion of “public sports service.” Thus, it is essential first to grasp the meaning of “public sports service” to elucidate “public sports services for rural older adults.” Since the term “public service” was incorporated into the government’s work report in 2002, there has been a surge in academic research regarding public sports services. However, two distinct designations persist: “sports public service” and “public sports service.” Scholars advocating for the term “sports public service” argue that this expression is more suitable, as it reflects an alternate usage of the term “public sports.” (8) Furthermore, from a structural linguistic perspective, “sports public service” has a simple structure and conveys meaning accurately, and it’s the only correct and standardized concept (9). Scholars who support “public sports service” contend that this designation more appropriately refers to public services within the sports domain. Proponents of the “public sports service” perspective assert its standardization stems from the usage patterns of the five major public utilities in China, which typically refer to “public education service,” “public health service,” and similar terms, all of which are widely recognized (10). The term “public sports service” is more relevant and appropriate in terms of the concepts of attributes, superordinate and subordinate concepts, the structural form of Chinese words, and the effectiveness of the use of similar words (11). Although there was some controversy at the beginning of the research over whether to use “sports public service” or “public sports service,” most scholars use “sports public service” and “public sports service” in their writings to refer to the same thing, and there is no essential difference between the two (12). In this article, we opt to use the term “public sports service,” aligning with the terminology employed in *the Thirteenth Five-Year Plan for Sports Development*, *the National Fitness Program (2016–2020)*, *the Outline for Building a Leading Sports Nation*, and various other policy documents.

Public sports service is an important part of China’s *13th Five-Year Plan for Promoting Equal Basic Public Services*, which together with education, housing security, healthcare, and other areas, constitute China’s basic public service content system. Currently, various interpretations of the concept of public sports services exist. From the public goods perspective, public sports services are defined as services that fulfill everyday societal needs and possess the characteristics of non-competitive and non-exclusive public goods (13). From the organizational function perspective, public sports services are viewed as functions designed to fulfill public sporting needs (11). According to the public interest perspective, public sports services encompass a range of activities to provide tangible and intangible goods to promote the public interest, utilizing government resources and authority (14). From the supplying body perspective, sports services rendered by entities such as the government, private enterprises, and the third sector qualify as public sports services (9). This study adopts a preference for the “public interest interpretation,” emphasizing that public benefit is both the starting point and the ultimate goal of public sports services, achievable solely through the realization of public interest (15). Following *the Report of the 19th National Congress of the Communist Party of China*, socialism with Chinese characteristics has entered a new era. The connotation of public sports services requires further expansion and enrichment. Moreover, addressing the general public’s increasingly diverse and multi-layered sports and fitness needs while ensuring equitable access to sports participation has become a fundamental responsibility and mission of public sports services in the new era. In conclusion, our study defines “public sports service” as various activities conducted by multiple stakeholders, primarily led by the government, to offer tangible and intangible resources to promote the public interest in sports, leveraging their respective resource advantages while adhering to a people-centered approach.

As mentioned earlier, the rural older adults in our study are those aged 50 and older who live in townships, market towns, and villages. Thus, public sports services for rural older adults are defined as a variety of actions or activities carried out by multiple government-led entities that make use of their respective resource advantages to realize public interests in sports and provide both tangible and intangible goods for rural older adults aged 50 and older living in townships and market towns while adhering to a people-centered approach. Its ultimate aim is to address the increasingly diverse and multi-faceted physical fitness needs of rural older adults. In the context of this concept, the various acts or activities provided primarily encompass the introduction of sports policies and regulations, the investment of funds in sports, the construction of sports venues and facilities, the organization of sports activities for older adults, the monitoring of physical fitness, the establishment of sound sports organizations for older adults, the training of sports cadres, and the provision of sports information and consulting services, among other initiatives.

### 3.2 Demand for public sports services among rural older adults

At present, there are only a few domestic studies that examine sports for seniors from the perspective of public services, the “demand for public sports services among rural older adults” has not yet been clearly defined. Some studies point out that the “demand for public sports services” is an economic activity realized by the public through the common consumption of public sports products related to their lifestyles and recreational activities (16). Others suggest that the “demand for public sports services” arises from various social groups pursuit of personal development and the need to meet their physical exercise requirements (17). According to Jiang Yuan (2015), the “demand for public sports services” can be defined as the aggregate of public sports goods or the volume of public sports services that residents acquire when engaging in physical exercise at a particular moment in public facilities (18). Combining these viewpoints and considering the need for research, our study regards the “demand for public sports services among rural older adults” as a condition of inadequacy resulting from the disparity between the level of sports services accessible to them and the level of services they aspire to receive within a specific time frame. The condition of inadequacy represents a prerequisite for developing public sports service demand among rural older adults. Willingness to obtain is the subjective need of rural older adults, including financial investment, construction of venues and facilities, construction of management systems, and guidance on activities. Should obtain is an unperceived objective need of rural older adults based on their health status, sports participation characteristics, healthy lifestyles, and other needs (Table 1).

**Table 1:** Statistical table of correlation between the content systems of public sports service demand.

Public sports service demand	D_1_	D_2_	D_3_	D_4_	D_5_	D_6_	D_7_	D_8_	D_9_
D_1_: Construction of sports facilities suitable for older adults	1.00								
D_2_: Establishment of organizations for physical exercise for older adults	0.593**	1.00							
D_3_: Guidance on participation in physical exercise for older adults	0.523**	0.685**	1.00						
D_4_: Organization of physical exercise activities suitable for older adults	0.470**	0.602**	0.644**	1.00					
D_5_: Health and fitness literacy for older adults	0.473**	0.500**	0.539**	0.615**	1.00				
D_6_: Physical fitness monitoring service for older adults	0.409**	0.427**	0.496**	0.525**	0.617**	1.00			
D_7_: Developing the backbone of senior sports	0.354**	0.545**	0.520**	0.489**	0.459**	0.420**	1.00		
D_8_: Advocacy and mobilization of older adults for participation in physical activity	0.402**	0.536**	0.541**	0.454**	0.494**	0.385**	0.658**	1.00	
D_9_: Institutional strengthening of sport for older adults	0.375**	0.512**	0.483**	0.461**	0.463**	0.380**	0.619**	0.693**	1.00
Mean	4.37	4.11	4.13	4.21	4.26	4.33	3.90	4.03	4.09
Standard deviation	0.86	0.93	0.93	0.86	0.85	0.89	1.03	0.97	0.97

*p < 0.05; **p < 0.01; ***p < 0.001.

## 4 Results and analyses

### 4.1 Characteristics of rural older adults’ demand for public sports services

As can be seen from combing through the relevant literature, most scholars focus on the six aspects of the public sports service system (sports information service, sports activities service, sports venues and facilities service, sports guidance service, sports organization service, and physical fitness monitoring service), and have further subdivided them into multiple specific demands for discussion and analysis (2, 5, 6, 19, 20), Some scholars have also taken into account the policy and legal guarantee elements (2, 5) and financial input guarantee elements (5, 21) of the public sports service system. Some scholars have found in their research that cultivating older adult sports leaders plays a significant role in establishing sports organizations, organizing sports activities, and promoting sports policies, thereby encouraging older adults to participate in physical exercise actively (2). To understand the specific content of rural older adults’ demand for public sports services and the extent of this demand, our study structured the demand of rural older adults for public sports services into nine aspects (see D_1_ to D_9_ in Table 2, which are labeled as “facilities,” “service organization,” “service guidance,” “activity content,” “fitness knowledge,” “monitoring service,” “backbone training,” “publicity and mobilization,” and “system construction”). The reliability test was conducted on the rural older adult public sports service demand scale composed of these nine aspects. The overall Cronbach’s *α* was 0.89, indicating that the scale has a high internal consistency. Meanwhile, the exploratory factor analysis showed that KMO = 0.83, *p* < 0.05, which was suitable for factor analysis.

**Table 2:** Statistical table of the impact of the characteristics of the population of rural older adults on the specific demand for public sports services.

Introducing variable	Model 1	Model 2	Model 3	Model 4	Model 5
	Facilities	Service organization	Service guidance	Activity content	Fitness knowledge
	OR	OR	OR	OR	OR
Sex (Ref = female)	1.078	0.966	1.083	1.041	1.035
Age (Ref = 70 years and under)					
60 years and under	1.500	0.990	1.099	0.909	1.134
60–70 years	1.643*	1.075	1.123	1.056	0.980
Region (Ref = Western)					
Eastern	0.711**	2.087*	2.087*	4.885**	1.041
Central	0.945	1.183	1.185	1.123	0.884
Marital status (Ref = single)	0.878	1.791**	1.076	1.121	0.604
Education (Ref = primary school and below)					
Senior high school and above	0.855	0.991	0.711	0.870	1.223
Junior high school	1.417	1.531	1.395	1.601*	1.802*
Living arrangement (Ref = other living arrangements)					
Live alone	2.650**	1.971*	2.046*	2.085*	1.933
Living with spouse/partner	2.882**	1.348	1.880*	2.173*	2.386**
Living with children	2.943**	2.303**	2.814**	2.154*	2.356**
Economic situation (Ref = more difficult)					
Affluent and above	1.151	1.752*	1.356	1.258	1.416
General	1.183	1.201	1.071	1.144	1.719**
Daily physical activity (Ref = less)					
More	0.563	0.768	0.785	0.908	0.916
General	0.919	0.956	1.321	1.178	1.224

Introducing variable	Model 6	Model 7	Model 8	Model 9	Model 10
	Monitoring service	Backbone training	Publicity and mobilization	System construction	Total requirement
	OR	OR	OR	OR	OR
Sex (Ref = female)	0.975	1.006	1.097	1.142	1.017
Age (Ref = 70 and under)					
60 years and under	1.036	0.998	1.275	1.127	1.335
60–70 years	1.233	1.416*	1.220	1.270	1.178
Region (Ref = Western)					
Eastern	9.416**	2.295**	1.910*	2.865**	2.907**
Central	0.777	0.778	0.722	1.748*	1.002
Marital status (Ref = single)	0.961	0.630*	1.003	1.072	0.146
Education (Ref = primary school and below)					
Senior high school and above	1.431	1.336	1.691*	1.279	1.042
Junior high school	1.724*	1.400	1.689**	1.516*	1.487
Living arrangement (Ref = other living arrangements)					
Live alone	0.855	2.051*	2.359**	2.524**	2.609**
Living with spouse/partner	1.175	1.924*	1.880*	2.030*	2.099*
Living with children	1.344	1.651	2.545**	2.424**	3.421**
Daily physical activity (Ref = less)					
More	0.671	0.953	0.707	0.777	0.655
General	0.865	1.143	0.936	1.049	0.937

*p < 0.05; **p < 0.01; ***p < 0.001.

#### 4.1.1 An analysis of the importance ranking of the content of public sports service needs of rural older adults

**Figure 1:**
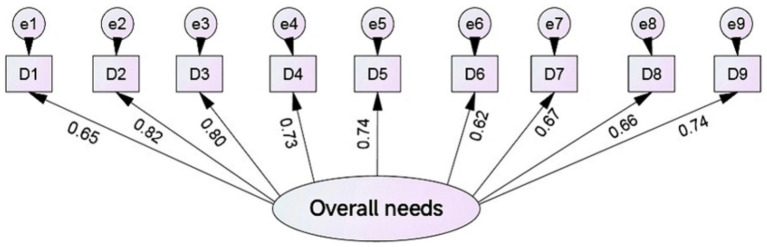
Validation of the content measurement model of public sports service demand of rural older adults.

By ranking the importance of the content of rural older adults’ public sports service needs, this process aids in prioritizing these needs, thereby enabling the efficient allocation of public sports resources. After conducting confirmatory factor analysis on the demand for public sports services among rural older adults, the results show (as shown in Figure 1) that the RMSEA of the measurement model was 0.031 (<0.05), and its goodness-of-fit index AGFI, CFI, NFI, and IFI were 0.94, 0.97, 0.93, and 0.98, respectively, all exceeding the standard of 0.90. Meanwhile, the composite reliability, CR, of the latent variables was 0.84. It shows that the model aligns well with the data obtained from the research, indicating good measurement validity. Regarding the standardized coefficient *β* values in the measurement model, the corresponding values for D_1_ to D_9_ are 0.65, 0.82, 0.80, 0.73, 0.74, 0.62, 0.67, 0.66, and 0.74, indicating that the variance of the explanation of the overall demand (β^2^) for D_1_ to D_9_ was 42.25, 67.24, 64.00, 53.29, 54.76, 38.44, 44.89, 43.56, and 54.76%, respectively. The ordered ranking of the nine major need components for rural older adults, in descending order, is as follows: ① Establishing organizations for physical exercise for older adults; ② Guiding older adults to participate in physical exercise; ③ Strengthening the system of sports for older adults; ④ Providing older adults with health and fitness knowledge; ⑤ Organizing sports and exercise activities suitable for older adults; ⑥ Cultivating the backbone of sports for older adults; ⑦ Publicizing and mobilizing older adults to participate in sports and exercise; ⑧ Constructing sports venues and facilities suitable for older adults; ⑨ Providing older adults with physical fitness monitoring service.

#### 4.1.2 Analysis of the correlation between the contents of the demand for public sports services among rural older adults

Overall, the nine domains of public sports service demand are closely related to each other (as shown in Table 1), and their degree of correlation is roughly divided into three levels:

(1) High correlations (r ≥ 0.6) are found between “service organization” and “service guidance” (0.685**), “service organization” and “activity content” (0.602**) with “service guidance” and “activity content” (0.644**), “activity content” and “fitness knowledge” (0.615**) with “fitness knowledge” and “monitoring service” (0.617**), “backbone training” and “publicity and mobilization” (0.658**), and “backbone training” and “system construction” (0.693**) with “publicity and mobilization” and “system construction” (0.693^**^). It shows that service organization is closely related to service guidance, and both have an important impact on activity content, fitness knowledge, and monitoring service; backbone training is closely related to publicity and mobilization, and both are closely related to system construction.
(2) The moderate correlation between service contents (0.45 ≤ r<0.60) is mainly reflected in the correlation between “facilities” and “service organization” (0.593**), “service guidance” (0.523**), “activity content” (0.470**), and “fitness knowledge” (0.473**); between “service organization” and “fitness knowledge” (0.500**), “backbone training” (0.545**), “publicity and mobilization” (0.536**), and “system construction” (0.512**); between “service guidance” and “fitness knowledge” (0.539**), “monitoring service” (0.496**), “backbone training” (0.520**), “publicity and mobilization” (0.544**), and “system construction” (0.483**); between “activity content” and “monitoring service” (0.525**), “backbone training” (0.489**), “publicity and mobilization” (0.454**), and “system construction” (0.461**); between “fitness knowledge” and “backbone training” (0.459**), “publicity and mobilization” (0.494**), and “system construction” (0.463**).
(3) The correlation between service contents is moderately low (0.30 ≤ r < 0.45), which is mainly reflected in the correlation between “facilities” and “monitoring service” (0.409**), “backbone training” (0.354**), “publicity and mobilization” (0.402**), and “system construction” (0.375**); between “service organizations” and “monitoring service” (0.427**); between “monitoring service” and “backbone training” (0.420**), “publicity and mobilization” (0.385**), and “system construction” (0.380**).

### 4.2 Analysis of factors influencing the content of demand for public sports services among rural older adults

Combined with existing studies and previous literature analyses, we found that the influencing factors on the demand for public sports services among rural older adults mainly include the demographic characteristics of the investigators, their health status, their individual lifestyles, and their awareness of physical activity. To further analyze the factors related to the specific demand for public sports services among rural older adults, our study introduced binomial logistic regression and concurrently categorized the degree of demand for public sports services among rural older adults based on the consistency of the degree of the nine needs (five types) and classified “very much in need,” “more in need” and “more in need” as well as “more in need” and “less in need,” “comparative need” and “general need” were grouped and named “need” (indicated by 1), “no need” and “no need at all” were grouped and named “need” (indicated by 1), and “no need” and “no need at all” were grouped. “No need” and “no need at all” are grouped as “no need” (denoted by 0) so that the five categorical variables of the degree of need become two categorical variables Y (0, 1). After this treatment, the nine needs become nine binomial logistic regression models obeying Y (0, 1).

#### 4.2.1 Influence of rural geriatric demographic characteristic variables on the specific content of demand for public sports services

Demographic characteristics refer to indicators that can reflect the socio-economic background of a group and the basic conditions of individuals, specifically including sex, age, region, cultural level, marital status, living arrangement, economic status, and other variables. Exploring the influence of demographic characteristics of rural older adults on the content of public sports service demand can help with precise policy-making and optimal allocation of public sports services resources. From the results of the analyses (as shown in Table 2), among the variables of rural geriatric demographic characteristics, sex and the amount of daily physical activity do not affect the degree of demand for the nine components of public sports services, while age, region, marital status, cultural level, economic status, and other variables have varying degrees of influence on the demand for public sports services, specifically:

The most important factor influencing the level of demand for public sports services among older adults is “living arrangement,” and except for “physical fitness monitoring service,” which is not affected by the living arrangement of older adults, the other eight services are all affected by their “living arrangement.” It is easy to find out from the odds ratio (OR) and taking “other living arrangements” as the reference, the proportion of older adults living alone who choose “need” for “facilities,” “service organization,” “service guidance,” “activity content,” “backbone training,” “publicity and mobilization,” and “system construction” is 2.650 times, 1.971 times, 2.046 times, 2.085 times, 2.051 times, 2.309 times, and 2.524 times that of the reference, respectively; the proportion of older adults living with their spouses/partners who choose “need” for “facilities,” “service guidance,” “activity content,” “fitness knowledge,” “backbone training,” “publicity and mobilization,” and “system construction” is 2.882 times, 1.880 times, 2.173 times, 2.386 times, 1.924 times, 1.880 times, and 2.030 times that of the reference, respectively; the proportion of older adults living with their children who choose “need” for “facilities,” “service organization,” “service guidance,” “activity content,” “fitness knowledge,” “publicity and mobilization,” and “system construction” is 2.943 times, 2.303 times, 2.814 times, 2.154 times, 2.356 times, 2.545 times, and 2.424 times that of the reference, respectively. It indicates that different living arrangements of older adults lead to different levels of family or social support, affecting their demand for public sports services.

The second most important factor influencing the level of demand for public sports services among older adults is “regional factor.” From the odds ratio (OR), and taking older adults in the “western region” as the reference, the proportion of older adults in the “eastern region” who hold the view of “needing” for the eight items of “facilities,” “service organization,” “service guidance,” “activity content,” “monitoring service,” “backbone training,” “publicity and mobilization,” and “system construction” is 0.711 times, 2.087 times, 2.087 times, 4.885 times, 9.416 times, 2.295 times, 1.910 times, and 2.865 times higher than that of the reference, respectively. It can be seen that older adults in the eastern region consider the demand for facilities and venue services less important than those in the western region, indicating that the facilities in the eastern region are relatively complete. However, the high multiplicity of choosing “very much need” in other aspects, such as “activity content” and “service guidance,” indicates that in the eastern region, people are more eager to improve the construction of sports services other than sports venues and facilities, and, in particular, to strengthen the work of “monitoring service,” which is 9.416 times more need in the eastern region than in the western region. There is no significant difference in the level of need for the eight sports services between older adults in the central region and older adults in the western region, but there is no difference in the level of need for “system construction.” The proportion of older adults in the “central region” who have a “need” for “system construction” is 1.748 times higher than that of the reference of older adults (western region). It shows that compared with the western region, institutional strengthening is an issue that needs to be addressed in the process of building public sports services for older adults in the central region.

The third important influencing factor of the demand for public sports services among rural older adults is “education” level. From the odds ratio (OR), and taking older adults at “primary school and below” education level as the reference, the proportion of older adults with “senior high school and above” education level who chose “need” in “publicity and mobilization” was 1.691 times higher than that of the reference; the proportion of older adults with “junior high school” education level who held the view of “need” in “activity content,” “fitness knowledge,” “monitoring service,” “publicity and mobilization,” and “system construction” is 1.601 times, 1.802 times, 1.724 times, 1.689 times, and 1.516 times higher than that of the reference, respectively. It indicates that older adults with a junior high school education or above have a significantly higher demand for public sports services than those with a primary school education or below. There are significant differences in the demand for “activity content,” “fitness knowledge,” “monitoring service,” “publicity and mobilization,” and “system construction” among rural older adults with different education levels, while the demand for “facilities,” “service organization,” “service guidance,” and “backbone training” is basically the same.

The fourth important influencing factor of the demand for public sports services among rural older adults is “economic situation.” From the odds ratio (OR), and taking older adults in “relatively difficult” economic families as the reference, the proportion of older adults from “affluent and above” economic families who held the view of “need” in “service organization” and “publicity and mobilization” is 1.752 times and 1.844 times higher than that of the reference, respectively; the proportion of older adults from “general” economic families who held the view of “need” in “fitness knowledge” and “backbone training” is 1.719 times and 1.609 times higher than that of the reference, respectively. It indicates that there are significant differences in the demand for public sports services among older adults with different economic statuses, and those with relatively better economic conditions have a higher demand for public sports services, and there is also a certain hierarchy. The influence of economic status on the demand for public sports services among older adults is only reflected in “service organization,” “publicity and mobilization,” “fitness knowledge,” and “backbone training.”

In addition, age and marital status also have an impact on the demand for public sports services among older adults. From the odds ratio (OR), and taking older adults aged “70 years and above” as the reference, the proportion of older adults aged “60–70 years” who held the view of “need” in “facilities” and “backbone training” is 1.643 times and 1.416 times higher than that of the reference, respectively; compared with older adults without a spouse as the reference, the proportion of older adults with a spouse who held the view of “need” in “service organization” and “backbone training” was 1.791 times and 0.630 times higher than that of the reference, respectively. It indicates that older adults of “lower age” have a higher demand for “facilities” and “backbone training.” Older adults with a spouse pay more attention to “service organization,” but less to “backbone training.”

#### 4.2.2 Influence of variables such as lifestyle, health status, and physical activity awareness on the specific content of demand for public sports services among rural older adults

Lifestyle refers to the behavioral performance and activity habits of rural older adults in their daily life, which is divided into five parts: smoking behavior, living arrangement, sedentary behavior, sports participation, and exercise behavior; health status is the overall physical, mental, and social adaptation of rural older adults, covering the number of chronic diseases, self-assessment of health, and self-care ability; the degree of physical activity awareness is the degree of understanding and recognition of the value, role, and benefits of physical activity by rural older adults. Physical activity awareness refers to the degree of understanding and recognition of the value, role, and benefits of physical activity among rural older adults and can be divided into two dimensions, namely, “physical fitness enhancement and disease prevention and control” and “mental health and achievement and interaction.” From the survey results (as shown in Table 3), the three variables of “smoking behavior,” “level of sports participation,” and “sports behavior” have no effect on the demand for the nine components of public sports services. The finding that the three variables of “smoking behavior,” “exercise participation level,” and “exercise behavior” have no effect on the demand for the nine contents of public sports services has indeed overturned the previous knowledge of many scholars, and it is worthwhile to reveal the reasons for this. On the other hand, the variables of sedentary behavior, perception of physical activity, personal health status, self-care ability, and sleep behavior have a broad and far-reaching impact on the demand for public sports services, specifically:

**Table 3:** Statistical table of the impact of older adults’ behavioral habits, exercise cognition and health status on the specific demand for public sports services.

Introducing variable	Model 1	Model 2	Model 3	Model 4	Model 5
	Facilities	Service organization	Service guidance	Activity content	Fitness knowledge
	OR	OR	OR	OR	OR
Smoking behavior (Ref = yes)	1.659	0.900	0.919	0.884	0.725
Sleep behavior (Ref = poor)					
(a) Well	0.388**	0.655	0.860	0.763	0.990
(b) General	0.600	0.688	1.067	0.913	1.428
Sedentary behavior (Ref = never)					
(a) Often	2.020*	1.294	1.737	2.880**	2.225*
(b) Sometimes	2.405**	2.108*	2.227**	3.389**	2.198**
(c) Occasionally	1.900*	1.613	1.835*	2.104**	1.989*
Type of chronic disease (Ref = no chronic diseases)					
(a) A chronic disease	0.757	0.681	0.994	0.896	0.788
(b) Two chronic diseases	0.783	0.748	0.811	1.098	0.895
(c) Three or more	1.363	1.0.61	0.778	1.421	1.267
Self-care (Ref = completely unable to care for themselves)					
Fully self-care	8.879**	6.250*	5.285*	5.673*	2.424
Partially self-care	7.461*	5.954*	4.463	7.878*	2.874
Health status (Ref = healthy)	0.636	0.648	0.600*	0.525*	0.466**
Physical fitness and disease prevention and control	0.916	1.286**	1.143	1.186*	0.988
Mental health and achievement interaction	1.329**	1.133*	1.119*	1.197**	1.168**
Level of participation in the campaign	0.997	0.989	0.985	1.001	0.987
Motor behavior (Ref = intentional)	1.056	10.585	9.538	10.996	0.902

Introducing variable	Model 6	Model 7	Model 8	Model 9	Model 10
	Monitoring service	Backbone training	Publicity and mobilization	System construction	Total requirement
	OR	OR	OR	OR	OR
Smoking behavior (Ref = yes)	0.690	0.839	0.845	0.894	0.715
Sleep behavior (Ref = poor)					
(a) Well	1.132	0.746	1.782*	1.070	0.698
(b) General	0.911	0.698	1.102	0.810	0.607
Sedentary behavior (Ref = never sedentary)					
(a) Often	1.445	0.841	1.210	2.047*	2.297*
(b) Sometimes	1.552	1.217	1.997*	2.121**	3.427**
(c) Occasionally	1.420	0.708	1.438	1.241	2.119*
Type of chronic disease (Ref = no chronic diseases)					
(a) A chronic disease	0.979	0.637*	0.950	0.840	0.625
(b) Two chronic diseases	0.907	0.680	0.790	0.662	0.681
(c) Three or more	1.024	1.007	0.656	1.136	0.774
Self-care (Ref = completely unable to care for themselves)					
Fully self-care	2.213	0.698	0.599	1.507	3.322
Partially self-care	2.426	0.591	0.503	1.218	2.846
Health status (Ref = healthy)	0.457**	0.832	0.870	0.823	0.432**
Physical fitness and disease prevention and control	0.961	1.149*	1.086	0.989	1.133
Mental health and achievement interaction	1.133*	1.018	1.064	0.995	1.075
Level of participation in the campaign	0.995	0.994	0.983	0.981	0.995
Motor behavior (Ref = intentional)	1.081	0.897	0.957	1.065	0.907

(1) In the process of quantifying “exercise behavior,” “regular physical activity (moderate-intensity exercise lasting more than 30 min at least three times a week)” was classified as “action participation,” and vice versa as “intention to participate”; (2) *p < 0.05; **p < 0.01; ***p < 0.001.

Sedentary behavior is the primary factor affecting the demand for public sports services among older adults. From the odds ratio (OR), and taking older adults who are “never” sedentary as the reference, the proportion of older adults who are “often” sedentary and hold the view of “need” in the four items of “facilities,” “activity content,” “fitness knowledge,” and “system construction” is 2.020 times, 2.880 times, 2.225 times, and 2.047 times higher than that of the reference, respectively; the proportion of older adults who are “sometimes” sedentary and hold the view of “need” in the seven items of “facilities,” “service organization,” “service guidance,” “activity content,” “fitness knowledge,” “publicity and mobilization,” and “system construction” is 2.405 times, 2.108 times, 2.227 times, 3.389 times, 2.198 times, 1.997 times, and 2.121 times higher than that of the reference, respectively; the proportion of older adults who are “occasionally” sedentary and hold the view of “need” in the four items of “facilities,” “service guidance,” “activity content,” and “fitness knowledge” is 1.900 times, 1.835 times, 2.104 times, and 1.989 times higher than that of the reference, respectively.

Perception of physical activity among older adults is the second important factor influencing the demand for public sports services among older adults. From the odds ratio (OR), for every one-unit increase in the score of “physical fitness and disease prevention and control,” the proportion of older adults who hold the view of “need” in the three items of “service organization,” “activity content,” and “backbone training” will increase to 1.286 times, 1.186 times, and 1.149 times, respectively. For every one-unit increase in the score of “Mental Health and Achievement and Interaction,” the proportion of older adults who hold the view of “need” in the six items of “facilities,” “service organization,” “service guidance,” “activity content,” “fitness knowledge,” and “monitoring service” will be increased to 1.329 times, 1.133 times, 1.119 times, 1.197 times, 1.168 times, and 1.133 times higher than that of the reference, respectively. It shows that the more profound rural older adults’ understanding of the role of sports in their mental health and achievement, the more they will be able to promote the demand for public sports services.

The ability of older adults to take care of themselves is the third important factor affecting the demand for public sports services among older adults. Taking “completely unable to take care of themselves” as the reference, the proportion of “fully self-care” older adults who hold the view of “need” in the four items of “facilities,” “service organization,” “service guidance,” and “activity content” will be 8.879 times, 6.250 times, 5.285 times, and 5.673 times higher than that of the reference, respectively; the proportion of “needy” older adults who hold the view of “need” in the three items of “facilities,” “service guidance,” and “activity content” will be 7.461 times, 5.954 times, and 7.878 times higher than that of the reference, respectively. It shows that the more rural older adults are able to take care of themselves, the stronger their demand for public sports services.

The health status of older adults also has a greater impact on the demand for public sports services among older adults. Taking “healthy” older adults as the reference, the proportion of “unhealthy” older adults who hold the view of “need” in the four items of “service guidance,” “activity content,” “fitness knowledge,” and “monitoring service” is only 0.600 times, 0.525 times, 0.466 times, and 0.457 times higher than that of the reference, respectively. It shows that the more “unhealthy” older adults are, the less importance they attach to the demand for public sports services.

In addition, the sleep status of older adults has an impact on the demand for sports services such as “facilities” and “promotion and mobilization.” Taking older adults with “poor” sleep as the reference, the proportion of older adults with “well” sleep who hold the view of “need” in the four items of “facilities” and “publicity and mobilization” is only 0.388 times and 1.782 times higher than that of the reference. It shows that older adults with better sleep do not attach importance to venues and facilities, and they think that “publicity and mobilization of older adults to participate in physical exercise” is more important.

At the same time, our study found that the impact of the type of chronic disease on the demand for public sports services was also minimal, which also overturned some of our previous perceptions because we found that the proportion of older adults with “one chronic disease” was only 0.637 times higher than the proportion of older adults with “no chronic disease” as the reference. The proportion of older adults with a “need” for “backbone training” was only 0.637 times that of older adults as the reference. It shows that there is a pressing priority to strengthen the publicity and practical application of the role of physical exercise in disease prevention, management, and rehabilitation.

### 4.3 Satisfaction of rural older adults with existing public sports services

#### 4.3.1 Use of existing public sports services by rural older adults

The survey on the satisfaction of rural public sports services is based on whether older adults have used or received public sports services in the past year. So before starting the survey, we analyzed the use of public sports services first, and we summarized the public sports services that have been used or received into seven items: D_1_, using the public sports venues and facilities; D_2_, receiving sports and fitness guidance; D_3_, participating in sports activities suitable for older adults; D_4_, receiving publicity and mobilization for older adults to participate in sports and fitness; D_5_, having participated in organizations for older adults to participate in sports and fitness; D_6_, receiving consultancy services on health and fitness knowledge; D_7_, receiving physical fitness monitoring service.

As shown in Table 4, the utilization rate of various public sports services is very low, the public sports service with the highest utilization rate is public sports venues, which only 38.6% of the respondents have used, five public sports services have a utilization rate of less than 20%, and those who have received sports and fitness guidance only account for 10.3%. It indicates that the proportion of older adults who have used or received public sports services for older adults over the past year is significantly lower than the proportion of older adults who have used or received public sports services for older adults, which may be due to the insufficient supply of public sports services for rural older adults on the one hand and the inconsistency between the supply of and demand for public sports services among rural older adults on the other hand.

**Table 4:** Statistics on the use of existing public sports services by older adults.

Usage	D_1_	D_2_	D_3_	D_4_	D_5_	D_6_	D_7_
Yes	38.6%	10.3%	18.7%	14.0%	12.9%	21.2%	13.5%
No	61.4%	89.7%	81.3%	86.0%	87.1%	78.8%	86.5%

#### 4.3.2 Overall satisfaction with public sports services for rural older adults

In this study, we used the Likert scale to measure the satisfaction of rural public sports services and divided each service into five categories according to the degree of satisfaction and assigning the corresponding scores, i.e., “very satisfied” = 5 points, “more satisfied” = 4 points, “generally satisfied” = 3 points, “dissatisfied” = 2 points, “not satisfied at all” = 1 point.

On the whole (as shown in Table 5), the overall satisfaction with public sports services for rural older adults ranges from “generally satisfied” to “relatively satisfied,” and the average value of satisfaction with the seven public sports services ranges from 3 to 4. Physical fitness monitoring service scored the highest at 3.86; public sports venues and facilities services had the second highest satisfaction rate at 3.78; and sports and fitness promotion and mobilization scored the lowest at 3.59. Combined with the use of the existing public sports services by rural older adults, we can find that there is an obvious phenomenon of “low use and high evaluation” in the public sports services for rural older adults. It means that although the actual use rate of most services is low, the overall evaluation of the quality of the services by those who have already used or received the relevant services is relatively high. It indicates that rural public sports services have a certain degree of acceptance in terms of quality, content, and effectiveness and that the services positively improve the quality of life and health of rural older adults.

**Table 5:** Satisfaction score of public sports services for rural older adults.

Variable	D_1_	D_2_	D_3_	D_4_	D_5_	D_6_	D_7_
Mean	3.78	3.77	3.66	3.59	3.73	3.71	3.86
Standard deviation	0.94	0.92	0.83	0.92	0.86	0.94	0.97

#### 4.3.3 Analysis of factors affecting satisfaction with public sports services for rural older adults

To deeply analyze how different factors affect the satisfaction of public sports services for rural older adults, our study introduces binomial logistic regression again and classifies the satisfaction of public sports services for rural older adults. Based on the consistency of the classification of the degree of satisfaction of the seven services, we grouped “very satisfied,” “relatively satisfied,” and “generally satisfied” into one category and named it “satisfied,” while “dissatisfied” and “completely dissatisfied” were grouped into another category and named it “dissatisfied.”

##### 4.3.3.1 Impact of the characteristics of the population of rural older adults on their use satisfaction

From the results of the analyses (as shown in Table 6), there is no difference in the satisfaction of older adults with the seven public sports services in terms of sex, marital status, and so on, but there are differences in the satisfaction with some of the public sports services in terms of different regions, literacy levels, age, economic status, and daily physical activities, to be more specific.

**Table 6:** Statistical table of the impact of the characteristics of the population of rural older adults on satisfaction with public sports services.

Introducing variable	Model 1	Model 2	Model 3	Model 4	Model 5	Model 6	Model 7
	Venue service	Guidance service	Activity program service	Publicity and mobilization service	Fitness organization service	Knowledge consulting service	Monitoring service
Sex (Ref = female)	0.898	0.667	0.875	1.178	1.158	1.185	2.007
Age (Ref = 70 and under)							
60 years and under	0.531*	0.976	0.956	0.481	0.557	0.611	0.433
60–70 years	0.657	1.356	1.008	0.467	0.746	0.488	1.082
Region (Ref = Western)							
Eastern	0.886	1.723	0.229**	1.377	1.647	1.486	1.381
Central	0.510*	1.214	1.332	5.071**	2.068	1.307	2.200
Marital status (Ref = single)	0.776	1.133	0.940	1.156	1.582	1.369	2.024
Education (Ref = primary school and below)							
Senior high school and above	1.195	1.180	0.411**	2.362*	1.605	0.775	0.999
Junior high school	1.894*	1.505	0.722	1.371	1.572	2.299	2.121
Economic situation (Ref = more difficult)							
Affluent and above	1.180	0.715	1.564	1.471	1.225	0.838	1.011
General	1.513	0.935	1.141	2.926*	2.625	1.878	0.906
Daily physical activity (Ref = less)							
More	1.655	4.128	1.301	1.055	1.805	0.954	10.418*
General	1.521*	2.143	0.895	1.497	0.804	1.327	1.066

Models 1–7 represent the satisfaction with the use of seven public sports services such as D_1_, D_2_, D_3_, D_4_, D_5_, D_6_, and D_7_, respectively, OR = “more satisfied”/“not very satisfied”; *p < 0.05; **p < 0.01; ***p < 0.001.

There is no difference in the satisfaction of older adults in different regions in four services: D_2_, receiving sports and fitness guidance; D_5_, having participated in organizations for older adults to participate in sports and fitness; D_6_, receiving counseling services on health and fitness knowledge; D_7_, receiving physical fitness monitoring service. However, there are significant differences between the three services: D_1_, using public sports venues and facilities; D_3_, participating in sports activities suitable for older adults; D_4_, receiving publicity and mobilization for older adults to participate in sports and fitness. Taking older adults in the “western region” as the reference, the proportion of older adults in the “central region” who are “satisfied” with the “venue service” was only 0.510 times higher than that of the reference; the proportion of older adults in the “eastern region” who are “satisfied” with the “activity program service” is only 0.229 times higher than that of the reference, but the proportion of older adults in the “eastern region” who are “satisfied” with the “publicity and mobilization service” is 5.071 times higher than that of the reference. It shows that the level of satisfaction with guidance services, fitness organization services, knowledge consulting services, and monitoring services is basically the same in different regions, but compared with the western region, older adults in the central region are dissatisfied with venue services; older adults in the eastern region are highly satisfied with the publicity and mobilization services, but dissatisfied with the services of activity programs.

There is no difference in the satisfaction of older adults with different literacy levels in four services: D_2_, receiving sports and fitness guidance; D_5_, having participated in organizations for older adults to participate in sports and fitness; D_6_, receiving consultancy services on health and fitness knowledge; D_7_, receiving physical fitness monitoring service. However, there was a significant difference in three services: D_1_, using the public sports venues and facilities; D_3_, participating in sports activities suitable for older adults; D_4_, receiving publicity and mobilization for older adults to participate in sports and fitness. Taking older adults with “primary school and below” education level as the reference, the proportion of older adults with “junior high school” education level who are “satisfied” with the “venue service” is 1.894 times higher than that of the reference; the proportion of older adults with “high school and above” education level who are “satisfied” with the “activity program service” is only 0.411 times higher than that of the reference, but the number of “satisfied” persons in the “publicity and mobilization service” is 2.362 times higher than that of the reference.

In addition, older adults of different ages, economic status, and daily physical activity only have some influence on sports services in a few programs, while there is almost no difference in satisfaction with the majority of services. The specific performance is as follows: taking older adults aged “70 years and under” as the reference, the proportion of older adults aged “60 years and under” who are “satisfied” only with “venue service” is only 0.531 times higher than that of the reference; taking older adults whose families are “more difficult” financially as the reference, the proportion of older adults with “general” family finances who are “satisfied” with the “publicity and mobilization service” is 2.926 times higher than that of the reference; taking older adults with “less” daily physical activity as the reference, the proportion of older adults with “general” daily physical activity who are “satisfied” with the “venue service” is 1.521 times higher than that of the reference.

##### 4.3.3.2 Impact of lifestyle, health status, and physical activity perceptions on satisfaction with use among rural older adults

From the results of the survey (as shown in Table 7), the smoking behavior, living arrangement, health status, and perception of physical activity of older adults have no effect on the satisfaction obtained during the use of the seven sports services, but there is a significant difference in the satisfaction of some of the sports services for older adults with different self-care behaviors, sleeping behaviors, sedentary behaviors, and levels of participation in sports, specifically:

**Table 7:** Statistical table of the impact of lifestyle, health status, and physical exercise cognition on satisfaction with public physical education services among rural older adults.

Introducing variable	Model 1	Model 2	Model 3	Model 4	Model 5	Model 6	Model 7
	Venue service	Guidance service	Activity program service	Publicity and mobilization service	Fitness organization service	Knowledge consulting service	Monitoring service
Smoking behavior (Ref = yes)	0.787	0.468	0.795	1.766	2.237	0.621	0.888
Sleep behavior (Ref = poor)							
(a) Well	1.236	0.412	0.321*	1.404	0.213	1.520	1.642
(b) General	0.652	0.217	0.377	0.369	0.150	0.710	2.026
Sedentary behavior (Ref = never sedentary)							
(a) Often	1.792	4.642	0.658	0.836	0.545	1.393	1.425
(b) Sometimes	1.715	6.874*	1.138	2.219	0.880	4.028	5.073*
(c) Occasionally	1.288	3.496	0.875	2.020	0.596	7.785*	6.522*
Living arrangement (Ref = other living arrangements)							
Live alone	0.626	1.334	1.775	1.431	0.872	1.964	2.262
Living with spouse/partner	0.686	0.869	2.140	1.383	0.471	1.312	1.063
Living with children	0.811	0.848	1.068	0.863	0.177	0.616	0.649
Type of chronic disease (Ref = three or more)							
(a) No chronic diseases	3.431*	1.410	1.373	0.759	4.435	4.831	0.210
(b) A chronic disease	1.704	0.296	1.021	1.314	1.770	1.888	0.173
(c) Two chronic diseases	0.401	0.260	1.058	0.732	2.212	2.010	0.192
Self-care (Ref = completely unable to care for themselves)							
Fully self-care	0.201	0.225	0.438	0.889**	0.913	0.841**	0.150
Partially self-care	0.190	0.420	0.512	0.813**	0.143	0.052	0.101
Health status (Ref = healthy)	0.600	0.633	2.238	0.717	0.204	1.578	2.149
Physical fitness and disease prevention and control	1.017	1.294	0.865	1.263	0.776	1.054	0.948
Mental health and achievement interaction	0.959	1.241	1.121	0.896	2.626**	1.329	1.022
Level of participation in the campaign	1.036**	0.999	1.040*	1.037	0.761	1.037	1.041

Models 1–7 represent the satisfaction with the use of seven public sports services such as D_1_, D_2_, D_3_, D_4_, D_5_, D_6_, and D_7_, respectively, OR = “more satisfied”/“not very satisfied”; *p < 0.05; **p < 0.01; ***p < 0.001.

Sedentary behaviors among rural older adults have a greater impact on satisfaction with three types of physical education services: D_2_, receiving sports and fitness guidance; D_6_, receiving consultancy services on health and fitness knowledge; D_7_, receiving physical fitness monitoring service, for which there is no difference in satisfaction. Taking “never” sedentary older adults as the reference, the proportion of “sometimes” sedentary older adults who are “satisfied” with the “guidance service” is 6.874 times higher than that of the reference, while the proportion of older adults who are “satisfied” with the “monitoring service” is 5.073 times higher than that of the reference. The proportion of “occasionally” sedentary older adults who are “satisfied” with “knowledge consulting service” and “monitoring service” is 7.785 times and 6.522 times higher than that of the reference, respectively.

Rural older adults’ ability to take care of themselves has a great impact on the satisfaction of two types of sports services: D_4_, receiving publicity and mobilization for older adults to participate in sports and fitness; D_6_, receiving consultancy services on health and fitness knowledge. Taking older adults who can “completely unable to take care of themselves” as the reference, the proportion of older adults who can “completely self-care” and “satisfied” with “publicity and mobilization service” and “knowledge consulting service” is 0.889 times and 0.841 times higher than that of the reference, respectively, while the number of older adults who can “partially self-care” are “satisfied” in the “publicity and mobilization service” is 0.813 times higher than that of the reference. It can be seen that, in terms of the quality of sports services in these two types, older adults who can “fully self-care” and “partially self-care” feel less satisfied than those who are “completely unable to take care of themselves”.

There is a big difference in the satisfaction of rural older adults with different levels of participation in sports with “venue service” and “activity program service”; when the level of participation in sports is increased by one unit, the proportion of older adults who are “satisfied” with “venue service” and “activity program service” will increase to 1.036 times and 1.040 times of the original level. If older adults attribute physical activity to “mental health and achievement,” for every one-unit increase in this perception, the proportion of older adults who are “satisfied” with “fitness organization services” will increase to 2.626 times the original level. It shows that improving the organization of fitness services is very beneficial for improving the perception of exercise awareness. Taking older adults suffering from “three or more” chronic illnesses as the reference, the proportion of older adults with “no chronic disease” who are “satisfied” with the “venue service” is 3.431 times higher than that of the reference. The reason why older adults with no chronic disease are so satisfied with the venue service but did not receive the same opinion from people with multiple chronic diseases needs to be further explored. Taking older adults with “poor” sleep as the reference, the proportion of older adults with “well” sleep who are “satisfied” with the “activity program service” is only 0.321 times higher than that of the reference. From the OR, we can find that the poor sleepers have less demands on the quality of the services provided by the activity program, which such an outcome would not have happened.

## 5 Conclusion and recommendations

### 5.1 Strengthening the awareness of needs and guiding older adults to participate in public sports services actively

Public sports services aim to meet the growing demand for physical fitness and improve people’s views on access, fairness, and happiness. To achieve this goal, rural older adults must see public sports services as an important part of their lives and keep a high demand for these programs. Survey results show that 30.6% of rural older adults in China still do not want public sports services. Some scholars think that due to the influence of traditional culture, such as the health view that values medicine over sports and the misconception that housework is equivalent to exercise, the majority of Chinese people do not attach sufficient importance to public sports services (22). Also, some scholars think that not having enough sports groups for older adults and not enough trained staff makes it hard for society to help older adults notice the importance of public sports services (23). Some researchers say that not paying enough attention to the need for sports services for older adults is due to not having enough investment from the government and society in these services. These points help explain the low demand for public sports services among rural older adults in China from different angles. However, looking closer at rural areas shows that the main barriers to increasing demand for sports services for older adults may be the area’s economic development and education level. From a theoretical point of view, Maslow’s Hierarchy of Needs says that people’s needs depend on the level of economic, cultural, and educational development in their country. In simple terms, when a region has high economic, cultural, and educational levels, people’s basic needs are met, and they start to have higher demands in life (24). Right now, China’s per capita GDP has gone over $10,000, which suggests that people will want more sports and fitness, leading to more varied needs for fitness methods, places, services, and experiences. However, the current situation is not good. According to Premier Li Keqiang’s remarks at the Third Session of the Thirteenth National People’s Congress in 2020, approximately 600 million people in China earn only 1,000 yuan a month. This means many people still face survival problems, especially since most rural older adults have low education levels, and many low-income and low-educated people live in rural areas. According to Marx’s theory of needs, human needs can be divided into three stages: survival needs at the first stage, enjoyment needs at the second stage, and development needs at the highest stage. So, for this group, the need for sports services can only become important after their survival needs are met. From a practical point of view, the study found that higher literacy levels mean a greater demand for public sports services. It also found that wealthy older adults care more about sports than those who are struggling. It is worth noting that in addition to cultural level and economic status, the demand for public sports services among rural older adults is also significantly related to factors such as age, region, marital status, sedentary behavior, and exercise participation. Therefore, in the process of establishing a demand-oriented public sports service supply, it is necessary to not only attach some importance to these factors in ideology but also objectively understand the demand of public sports service among rural older adults those who are middle-aged and high-aged, those in the western region, those without spouses, those with sedentary lifestyles, and those who are “non-active participants.” And increase publicity and guidance for them in action to stimulate their demand for public sports services. Specifically, county sports departments should cooperate with village committees, making full use of village radio, bulletin boards, WeChat groups, and other channels to publish knowledge of sports and fitness and information on activities regularly, to enhance older adults’ awareness of the benefits of physical exercise. At the same time, incentive mechanisms should be set up, such as the establishment of “health star” and other selection activities, to honor older adults who actively participate in physical exercise and to stimulate their inherent motivation to continue to participate.

### 5.2 Focusing on the relevance, importance, and impact of the content of demand, and clarifying the subjective demand picture of rural older adults

The people-centered view of sports development is the value orientation and fundamental purpose of sports development in China in the new era. To effectively improve the accuracy of public sports services for rural older adults, it is necessary to thoroughly understand the most important, most realistic, and most critical needs of rural older adults. The study found that there is an obvious correlation between the demands of public sports services for rural older adults, which can be divided into three levels: highly correlated, moderately correlated, and moderately to slightly correlated. Specifically, the three service contents of “service organization,” “service guidance,” and “service activities” are highly correlated with each other. The three service components of “backbone training,” “publicity and mobilization,” and “system construction” are highly related to each other as well. “Backbone training” and “publicity and mobilization” are moderately related to “system construction,” “service guidance,” “activity content,” and “fitness knowledge.” However, the correlation between “monitoring service” and other service content is relatively low. Given the above situation, the supply of rural public sports services cannot meet individual needs in isolation. It is recommended that county sports departments focus on the organic combination and coordination of service content and launch integrated service packages such as “sports organization-guidance-activities.” Meanwhile, by integrating the training of grassroots backbone personnel with publicity and mobilization, the standardization and institutionalization of service content should be promoted. Additionally, given the low relevance of monitoring services, service methods should be optimized separately to improve service accuracy.

In terms of the priority order of public sports service demands among rural older adults, the top three services that they most urgently want to improve are sports organization services, sports guidance services, and sports system construction services. This indicates that in the investment of rural public sports services, these three aspects should be given priority for development. Specifically, in terms of sports organization construction, the legalization and standardization of rural sports associations for older adults should be promoted, policy and financial support should be provided, and the vitality and service capabilities of grassroots sports organizations should be enhanced. In terms of sports guidance services, professional sports instructors should be regularly organized to visit rural areas to provide scientific and professional fitness guidance to older adults, to solve the problem of the lack of sports knowledge and skills for rural older adults. In terms of sports system construction, stable service supply guarantee policies should be introduced, and long-term service plans and supervision mechanisms should be clarified to avoid interruptions and arbitrariness in sports services, to enhance the trust and participation of rural older adults.

This study found that there are statistically significant differences in the specific demand of older adults for public sports services among demographic factors such as different regions, cultural levels, and economic conditions. Specifically, there are significant differences in the demand for public sports services among older adults in different regions. Although there are significant differences in system construction between the central and western regions, older adults in the eastern region have a lower perception of the importance of venue and facility demands than those in the central and western regions, but their demand for the other eight sports service contents is much higher than that of the central and western regions. In other words, rural older adults in the eastern region generally have a stronger willingness to demand public sports services. However, in terms of health status, the proportion of older adults in good health and essential health in the eastern region is 86.05%, significantly higher than 81.07% in the central region and 81.22% in the western region (25). Furthermore, the objective demand for sports services among rural older adults in the central and western regions is, in general, higher. Therefore, how to transform the objective demands of rural older adults in the central and western regions into subjective demands should be given attention. It is suggested that county sports departments formulate differentiated policy measures based on regional differences, with a particular focus on strengthening publicity and guidance in the central and western regions, gradually realizing the transformation from objective demands to subjective demands. In addition, in terms of older adults’ lifestyles and exercise awareness, factors such as sedentary behavior, living arrangements, awareness of physical exercise, and personal health status are significantly correlated with rural older adults’ demand for public sports services. Among them, the top three influencing factors are the living conditions of older adults, sedentary behavior, and exercise cognition. It shows that we should pay more attention to these three influencing factors to improve the precision of public sports services for rural older adults. Of course, in addition to depicting the subjective demand picture of rural older adults for sports services, it is also necessary to explore the problems between subjective demands and objective conditions and then actively improve them to enhance people’s sense of gain and happiness.

### 5.3 Focusing on differences in the use and satisfaction of public sports services, and promoting the precise supply of public sports services

The data on the usage rates and satisfaction levels of public sports services serve as essential references for assessing the supply situation of sports services for rural older adults. They also provide critical foundations for enhancing the input efficiency of various public sports service resources and formulating policies for older adults. According to the survey results, the usage rates of seven sports services—such as sports venues and facilities, sports fitness guidance, and sports fitness activities—are relatively low, with the highest usage rate not exceeding 40% and most rates falling below 20%. Based on the on-site investigation, the primary reasons can be categorized into three main aspects. First, the publicity and dissemination rate of sports fitness services is low. Many older adults report that they lack clarity regarding the specific offerings of sports services and often feel confused when attempting to utilize them. Many are drawn to the novelty of sports services but fail to recognize their value and functionality. In response to this, county sports departments should work with village committees to emphasize the value and function of sports services and raise awareness among older adults through door-to-door publicity and the establishment of publicity boards. Second, the supplied content does not align with the actual demand of older adults. Rural older adults primarily select activities such as “walking, mountain climbing, or running” for exercise and exhibit a greater demand for sports fitness guidance and sports organization services. They demonstrate relatively low demand for sports venues and facilities, as evidenced by the order of demand. In reality, resources allocated to rural public sports services are primarily invested in sports venues and facilities, resulting in inadequate support for the development of alternative services. This situation leads to a lower frequency of use or acceptance of other types of public sports services among older adults. In response to this, county sports departments should adapt to local conditions, further optimize resource allocation, formulate specific plans for the proportion of funding and personnel input, and prioritize limited resources for sports fitness guidance, sports organization services, and other content provisions to meet actual demands. Third, traditional beliefs and concepts discourage rural older adults from intending to or finding time to engage with sports services. Many assume that rural older adults have ample time to engage with sports services. However, in reality, rural older adults, particularly those facing challenging economic conditions, continue to fulfill various family responsibilities, spending the majority of their time on farming, household chores, and caring for grandchildren, which leaves them with minimal leisure time for sports and fitness activities. From their perspective, ensuring material wellbeing and raising the next generation take precedence over pursuing personal development, resulting in infrequent use of sports services. In response to this, county sports departments should provide sports services flexibly based on the actual situation of busy and idle farming seasons. For example, they can hold regular fitness and leisure activities during the idle season and provide relevant support measures to increase the actual participation rate of rural older adults.

The survey results regarding the satisfaction of rural older adults with public sports services indicate that several factors influence this satisfaction. Specifically, region, culture, and sedentary behavior affect the satisfaction of three aspects of public sports services. Additionally, self-care ability and sports participation influence two aspects of satisfaction, while age, economic status, daily physical activity, types of chronic diseases, health status, and sleep quality significantly affect only one aspect. Regarding depth, the satisfaction of rural older adults with sports venue services is influenced by the most significant number of factors, totaling six. This is followed by activity services and publicity services, each influenced by four factors. The satisfaction of sports knowledge consultation services is affected by two factors, while other services are influenced by only a single factor. Undoubtedly, the broader the influence of a particular factor on public sports services for rural older adults, the more attention it should receive from the government, necessitating corresponding measures to address it. The more factors that affect a certain content of public sports services, the more it needs to be planned comprehensively, considering the coordination of multiple influencing factors to better improve the satisfaction of that content. Therefore, to effectively improve the satisfaction of public sports services for rural older adults, county sports departments should formulate special improvement plans for sports venues, activity services, and publicity services, which are affected by many factors, to better meet the actual demands of rural older adults and gradually improve overall satisfaction levels.

In summary, this study reveals the multidimensional characteristics of rural older adults’ demand for public sports services in China and identifies several crucial associated variables, including demographic variables such as living arrangements and region, as well as lifestyle, exercise awareness, and health status. China’s approach shows that increasing the level of participation in sports among rural older adults depends on systematic public support and policy intervention, rather than solely on market mechanisms or community self-organization. Therefore, this study not only contributes to the theoretical and practical construction of the sports service system for rural older adults in China but also provides policy references and action inspirations for the international community, especially for countries with insufficient coverage of public sports services. Although this study made comprehensive arrangements in terms of sample coverage, scale design, and statistical analysis, there are still certain limitations. First, the survey data are mainly based on self-reported information from respondents, which may be subject to subjective judgment and memory bias. Second, certain groups that were unable to participate in the survey (such as those who were dissatisfied or did not use the service) may be underrepresented in the results. In addition, because the study used a cross-sectional design, it was difficult to reflect the dynamic process of demand changes over time. Owing to limitations in data availability, some important contextual factors that may influence demand and satisfaction (such as accessibility of sports facilities, participation in previous fitness programs, etc.) were not included in the model. Future research can build on this study by introducing longitudinal tracking designs, mixed research methods, or on-site behavioral observation tools to gain a more comprehensive and dynamic understanding of the public sports service demand mechanisms of rural older adults, to provide a basis for more accurate policy-making and service optimization.

## Data Availability

The raw data supporting the conclusions of this article will be made available by the authors, without undue reservation.
